# Global Burden of Lip and Oral Cavity Cancer From 1990 to 2021 and Projection to 2040: Findings From the 2021 Global Burden of Disease Study

**DOI:** 10.1002/cam4.70957

**Published:** 2025-05-10

**Authors:** Mingxing Chen, Jiangxi Li, Wei Su, Junming Huang, Chuanzhen Yang, Rui Li, Gang Chen

**Affiliations:** ^1^ Medical Research Public Service Center, The First Affiliated Hospital, Jiangxi Medical College Nanchang University Beijing China; ^2^ School of Life Science Jiangxi Science &Technology Normal University Nanchang China; ^3^ The Orthopedic Hospital, The First Affiliated Hospital, Jiangxi Medical College Nanchang University Nanchang China; ^4^ Department of Sports Medicine, Orthopedic Hospital, The First Affiliated Hospital, Jiangxi Medical College Nanchang University Nanchang China; ^5^ The Key Laboratory of Spine and Spinal Cord Diseases of Jiangxi Province Nanchang China; ^6^ Postdoctoral Innovation Practice Base, The First Affiliated Hospital, Jiangxi Medical College Nanchang University Nanchang China; ^7^ NHC Key Laboratory of Systems Biology of Pathogens, State Key Laboratory of Respiratory Health and Multimorbidity, National Institute of Pathogen Biology Chinese Academy of Medical Sciences & Peking Union Medical College Beijing China; ^8^ State Key Laboratory of Food Science and Technology Nanchang University Nanchang China

**Keywords:** epidemiology, global disease burden, lip and oral cavity cancer, spatiotemporal trends

## Abstract

**Background:**

The aim of this study was to estimate the global burden of lip and oral cavity cancer (LOC) and its trends in different genders, age groups, regions, and countries globally.

**Methods:**

Data were sourced from the Global Burden of Disease 2021 study.

**Results:**

During the 32‐year period, a 92.92% and 113.94% increase was estimated in the absolute counts of LOC deaths and disability‐adjusted life years (DALYs), respectively. Throughout the 32‐year period, males exhibited higher age‐standardized rates (ASRs) of incidence (ASIRs), prevalence (ASPRs), mortality (ASMRs), and DALYs (ASDRs) related to LOC. The age group of 60–64 years consistently recorded the highest numbers of new and prevalent cases across the years 1990, 2019, and 2021. In 2019 and 2021, the highest ASMR and ASDR were observed in individuals aged 95 years and older. Regions with low‐middle and low socio‐demographic index (SDI) consistently showed higher ASMRs and ASDRs associated with LOC from 1990 to 2021. Eastern Europe, South, North, and Southeast Asia exhibited a concentration of countries with higher ASIRs, ASPRs, ASMRs, and ASDRs in 2021. South Asia maintained high levels of ASIRs, ASPRs, ASMRs, and ASDRs in 2021. In 2021, Palau recorded the highest ASIR, ASPR, ASMR, and ASDR, followed by Pakistan. Projections indicate that ASIR, ASPR, ASMR, and ASDR are expected to increase by 7.40%, 10.10%, 2.85%, and 4.60%, respectively, from 2021 to 2040.

**Conclusion:**

LOC remains a critical public health concern that requires immediate attention, particularly among certain demographics such as males, aged 60–64 or 95 and older, as well as in low‐ and middle‐SDI regions, particularly Eastern Europe, South Asia (notably Pakistan), North Asia, and Southeast Asia.

## 1 Introduction

The lip and oral cavity cancer (LOC) is the most prevalent malignant neoplasm of the head and neck [1]. In 2020, it was estimated that there were 377,713 new cases and 177,757 deaths globally due to LOC [2]. LOC is more frequently diagnosed in men and older populations, with a notably higher mortality rate among women; its incidence also varies significantly according to socioeconomic factors [1]. Notably, only 40%–50% of individuals diagnosed with LOC survive for 5 years post‐diagnosis [3]. Consequently, LOC constitutes a substantial public health challenge worldwide. Previous studies have predominantly addressed the disease burden of LOC over the past few decades [4, 5, 6, 7, 8, 9, 10, 11, 12]. However, most of these investigations have been limited to specific countries or regions [4, 6, 7, 9, 10]. While a few studies have been conducted on a global scale, they are now somewhat outdated and in need of reevaluation [5, 8, 12].

This systematic analysis aimed to update and document the trends and more measurement variables related to LOC, specifically focusing on four key measures: incidence, prevalence, mortality, and disability‐adjusted life years (DALY). Additionally, it also examined four types of age‐standardized rates (ASRs): age‐standardized rate of incidence (ASIR), prevalence (ASPR), mortality (ASMR), and DALY (ASDR), utilizing Global Burden of Disease (GBD) data from 1990 to 2021. This study analyzed the global incidence, prevalence, mortality, and DALY of LOC, disaggregating the data by subgroups such as sex, age, geographic location, and nation, based on the estimates from the GBD 2021 study. We also investigated the spatial–temporal patterns and trends from 1990 to 2021. Lastly, we projected the disease burden for the next 20 years. The findings of this study are intended to provide valuable insights for health professionals and policymakers in the development and implementation of effective public health strategies to mitigate the substantial burden of this disease.

## 2 Materials and Methods

### 2.1 Overview of GBD

In this study, the data of incidence, prevalence, mortality, and DALYs of LOC were obtained from the Global Burden of Diseases, Injuries, and Risk Factors Study (GBD) 2021. The GBD 2021 evaluates health risks associated with 371 diseases and injuries, as well as 88 risk factors, across 204 countries and territories [13]. All estimates in this study are stratified by sex, age, location, and nation, covering the period from 1990 to 2021. Four spatial divisions were used to characterize the entire population features and identify countries or regions that necessitate targeted intervention of LOC. The first division is global, while the second is based on the socio‐demographic index (SDI) [14], which categorizes the world into five super regions: low SDI, low‐middle SDI, middle SDI, high‐middle SDI, and high SDI [15]. By comparing the SDI values across different countries and regions, researchers and policymakers can gain insights into the overall socioeconomic development and health status of various regions. The third division is based on geographical regions, consisting of 21 categories determined by epidemiological similarities and geographical proximity [16]. The fourth category encompasses 204 countries or regions [17].

### 2.2 Incidence, Prevalence, Mortality, and DALY Estimates

For the analysis of the all‐age population, we utilized data on the incidence, prevalence, mortality, and DALY estimates, along with ASIR, ASPR, ASMR, and ASDR per 100,000 population, as provided by the GBD 2021 study. For specific age cohorts (e.g., < 5 years, 5–9 years, and up to 95+ years), we employed crude rates, as GBD 2021 only supplies these for discrete age groups. All data are accessible online through the GBD results tool (http://ghdx.healthdata.org/gbd‐results‐tool). The estimates were generated stratified by sex (female and male) and across 20 age groups from birth to 95 years and older for 204 countries and territories, including subnational estimates for 21 GBD regions and 5 SDIs. The GBD 2021 dataset provides annual estimates spanning from 1990 to 2021.

### 2.3 Estimated Annual Percentage Change (EAPC) and Percentage Change

The analysis of the burden of disease for LOC utilized the number of new cases, total cases, deaths, and DALYs, alongside ASIRs, ASPRs, ASMRs, and ASDRs, each accompanied by 95% uncertainty intervals (UIs). ASRs were calculated by standardizing to the global age structure [18], thereby ensuring comparability across populations from different geographical locations and time periods. EAPC is an effective and widely utilized metric that has been extensively employed in prior research to monitor trends in indicators such as prevalence and incidence rates over specified timeframes [19]. The calculation of EAPC involves fitting a regression model to the natural logarithm of the rates, with time serving as a variable; the natural logarithm of each observation is fitted to a straight line, and the EAPC is determined based on the slope of this line [19]. A negative EAPC indicates a downward trend, while a positive EAPC signifies an upward trend [6]. The 95% confidence intervals (CIs) for the EAPC are derived from this fitted model. Furthermore, percentage change was also employed to quantify variations in the prevalence, incidence, mortality, and DALYs for the years 2019 and 2021 in comparison to the year of 1990.

Utilizing estimated EAPC values, hierarchical cluster analysis was conducted to evaluate the changing patterns of disease burden across different regions, aiming to identify regions that exhibit similar changes in disease burden [20]. The 21 regions were subsequently classified into four distinct categories: significant increase, minor increase, remained stable or minor decrease, and significant decrease [20]. Projections of future disease burden from 2022 to 2040 were generated using the Nordpred R statistical software package [21].

Data cleaning, computation, and graph plotting were performed using R software (version 4.3.1). Visualizations were produced with the ggplot2 package, while final graphical editing was carried out using Adobe Illustrator (version 24.0). The flow chart of this study was shown in Figure S1.

### 2.4 Data Sharing Statement

The data utilized for the analyses are publicly accessible through the Institute for Health Metrics and Evaluation (http://www.healthdata.org/; http://ghdx.healthdata.org/gbd‐results‐tool).

## 3 Results

### 3.1 The Global Burden of LOC in 1990, 2019, 2021 and During 1990 and 2021

In 2021, there were a total of 421,577 new cases of LOC globally, with a 95% UI ranging from 338,000 to 401,000. The global ASIR was 4.88 per 100,000 individuals (95% UI, 4.52–5.20). The total number of prevalence cases reached 1,538,008 worldwide, corresponding to a global ASPR of 17.71 per 100,000 (95% UI, 16.52–18.81). The estimated number of deaths attributed to LOC was 208,379 (95% UI, 191,288–224,162), corresponding to an ASMR of 2.42 deaths per 100,000 (95% UI, 2.23–2.60). In 2021, LOC was responsible for 5,664,617 DALYs (95% UI, 5,237,978–6,054,498) (Table S1).

A critical finding was the observed 142.18% increase in the incidence of LOC cases between 1990 and 2021. The ASIR of LOC increased by 14.29%. Over the 32 years analyzed, the absolute counts of LOC‐related mortalities and DALYs were estimated to have increased by 92.92% and 113.94%, respectively. In contrast, the ASMR and ASDR experienced slight decreases of 1.22% and 2.25%, respectively (Table S1).

**FIGURE 1:**
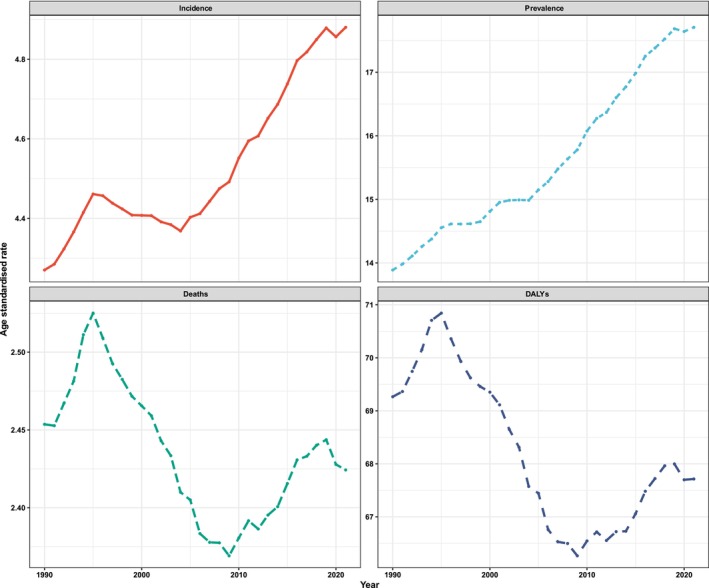
Time trends of the incidence, prevalence, mortality, and DALY of lip and oral cavity cancer globally from 1990 to 2021. The ASPR exhibited a consistent upward trajectory over the 32‐year period. The trends in ASIRs, ASMRs, and ASDRs can be summarized as follows: An initial increase was observed, with ASIRs and ASDRs rising from 1990 to 1995 and ASMRs escalating from 1991 to 1995. This initial rise was subsequently followed by a reduction, characterized by a decline in ASIRs between 1995 and 2004, along with decreases in ASMRs and ASDRs from 1995 to 2009. Thereafter, a significant increase occurred, with ASIRs rising from 2004 to 2019 and ASMRs and ASDRs increasing from 2009 to 2019. From 2019 to 2020, a decline was observed across ASIRs, ASPR, ASMRs, and ASDRs. Notably, the downward trends in ASMR and ASDR continued into 2021. ASDR, age‐standardized disability rate; ASIR, age‐standardized incidence rate; ASMR, age‐standardized mortality rate; ASPR, age‐standardized prevalence rate.

The temporal trends of ASIRs, ASPRs, ASMRs, and ASDRs for global LOCs from 1990 to 2021 were illustrated in Figure 1. The ASPR exhibited a generally increasing trend over the 32‐year period. The trends of ASIRs, ASMRs, and ASDRs can be summarized as follows: initially, an increase was observed, with ASIRs and ASDRs rising from 1990 to 1995, and ASMRs increasing from 1991 to 1995. This period of growth was followed by a decline, with ASIRs decreasing from 1995 to 2004, and both ASMRs and ASDRs declining from 1995 to 2009. Subsequently, a notable resurgence occurred, with ASIRs rising from 2004 to 2019, and ASMRs and ASDRs increasing from 2009 to 2019. Additionally, a decline in ASIR, ASPR, ASMR, and ASDR was observed during the period from 2019 to 2020. Furthermore, the decreasing trends in ASMR and ASDR persisted into 2021.

**FIGURE 2:**
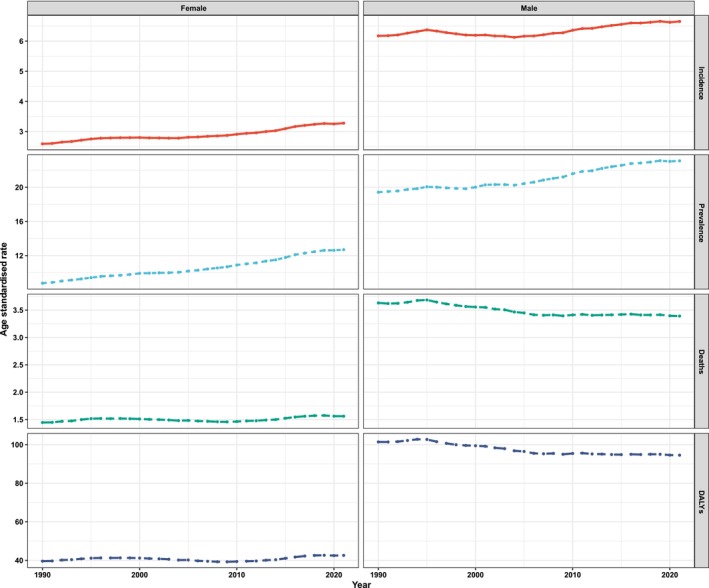
The trends of the incidence, prevalence, mortality, and DALY of different genders of lip and oral cavity cancer from 1990 to 2021. During the period from 1990 to 2021, males consistently demonstrated higher numbers and age‐standardized rates (ASIRs, ASPRs, ASMRs, and ASDRs) in comparison to females. ASDR, age‐standardized disability rate; ASIR, age‐standardized incidence rate; ASMR, age‐standardized mortality rate; ASPR, age‐standardized prevalence rate.

**FIGURE 3:**
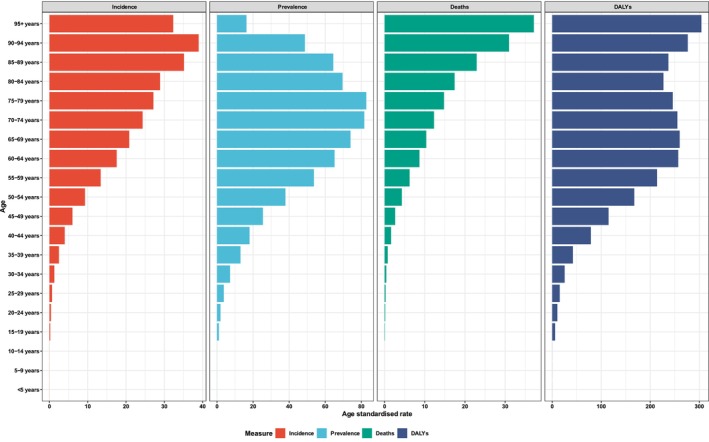
The global incidence, prevalence, mortality, and DALY of different age groups of lip and oral cavity cancer in 2021. In 2021, the highest ASIRs were observed in the age group of 90–94 years. The ASPRs at the age group of 75–79 were the highest in 2021. The highest ASMRs and ASDRs were recorded for individuals aged 95 years and older. ASIR, age‐standardized incidence rate; ASPR, age‐standardized prevalence rate.

In terms of gender stratification, males consistently exhibited higher numbers of cases, as well as higher ASIRs, ASPRs, ASMRs, and ASDRs compared to females during the period from 1990 to 2021 (Figure 2, Table S2). Notably, the ASMR increased by 7.59% for females while decreasing by 6.61% for males over these 32 years. Additionally, females demonstrated a greater variation in EAPC compared to males from 1990 to 2021 (Table S2). Figure 3 depicts the ASIRs, ASPRs, ASMRs, and ASDRs of global cases of LOC stratified by age group in 2021. The number of new and prevalent cases within the age range of 60–64 years was the highest across all age groups in 1990, 2019, and 2021 (detailed in Table S3). The highest ASIRs were observed in the age group of 90–94 years globally due to LOC for all 3 years—1990, 2019, and 2021. The highest ASPRs were recorded in the 75–79 age group during the same years. Moreover, the ASMR and ASDR were highest in individuals aged 95 years and older (ASMR highest: 1990, 2019, and 2021; ASDR: 2019, and 2021).

### 3.2 The Burden of LOC in Different Countries or Regions

**FIGURE 4:**
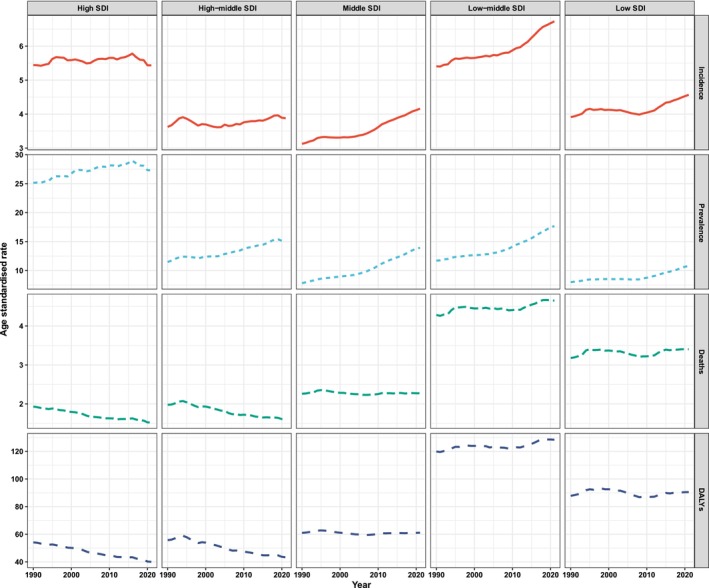
The trends of the incidence, prevalence, mortality, and DALY of different SDI locations of lip and oral cavity cancer from 1990 to 2021. From 1990 to 2021, high, and high‐middle SDI regions displayed a decreasing trend in ASMRs and ASDRs. The increase in ASIR and ASPR was more pronounced in middle and low‐middle SDI countries from 2010 to 2021. Furthermore, low‐middle and low SDI regions consistently reported higher ASMRs and ASDRs attributed to LOC throughout this period. In contrast, middle, low‐middle, and low SDI regions exhibited an increasing trend in ASMRs and ASPRs. Furthermore, both high and low‐middle SDI regions demonstrated the higher ASIRs and ASPRs over the 32 years. ASDR, age‐standardized disability rate; ASIR, age‐standardized incidence rate; ASMR, age‐standardized mortality rate; ASPR, age‐standardized prevalence rate; SDI, socio‐demographic index.

**FIGURE 5:**
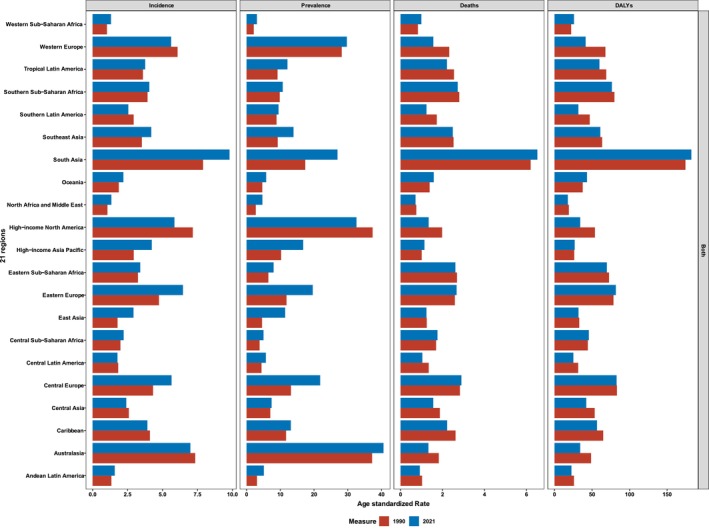
The incidence, prevalence, mortality, and DALY of 21 regions in GBD 2021 of lip and oral cavity cancer patients in 2019 and 2021. South Asia almost has the highest ASIRs, ASMRs, and ASDRs in the 21 GBD regions, while Australasia has the highest ASPRs in 2019 and 2021. ASDR, age‐standardized disability rate; ASIR, age‐standardized incidence rate; ASMR, age‐standardized mortality rate; ASPR, age‐standardized prevalence rate.

From 1990 to 2021, high and high‐middle SDI regions exhibited a decreasing trend in ASMRs and ASDRs (Figure 4, Table S4). Conversely, middle, low‐middle, and low SDI regions demonstrated an increasing trend in both ASMRs and ASPRs. Throughout this period, low‐middle and low SDI regions consistently recorded higher ASMRs and ASDRs attributed to LOC (Figure 4). Specifically, the low‐middle SDI region experienced the largest increases in mortalities and DALYs, with an EAPC for mortalities of 0.20 (95% CI: 0.14–0.26) and for DALYs of 0.17 (95% CI: 0.12–0.22). Furthermore, high and low‐middle SDI regions exhibited higher ASIRs and ASPRs during the 32‐year period. In contrast, high and high‐middle SDI regions displayed the most substantial decreases in mortalities and DALYs, characterized by the lowest age‐standardized EAPCs during this timeframe (Table S4). The ASIRs, ASPRs, ASMRs, and ASDRs for 2021 are illustrated in Figure 5, which presents data for the 21 regions included in the GBD study. South Asia consistently had the highest numbers of new cases, prevalent cases, mortalities, and DALYs among these 21 regions in 1990, 2019, and 2021 (Table S5). Additionally, South Asia recorded the highest ASIRs, ASMRs, and ASDRs within these regions, while Australasia reported the highest ASPRs across the same years.

**FIGURE 6:**
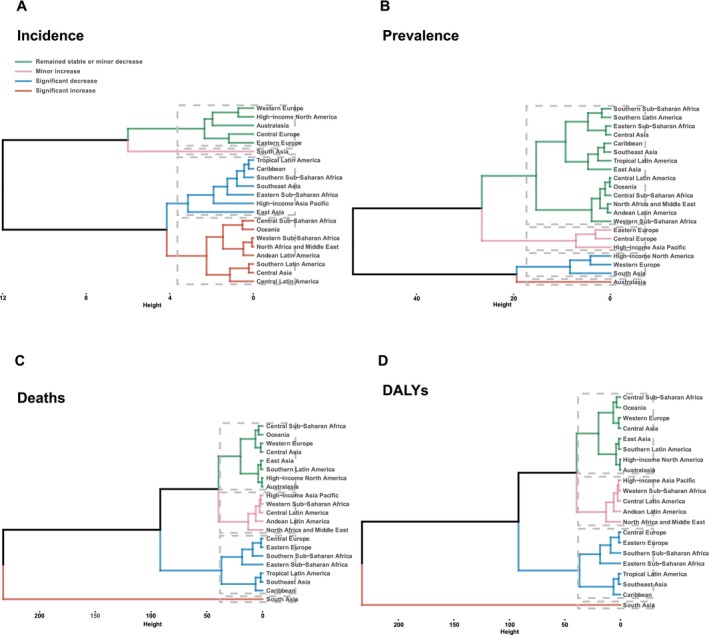
The cluster analyses the incidence, prevalence, mortality, and DALY of lip and oral cavity cancer using the clustering analysis during 1990 and 2021. Lines in the same color represent the same increase/decrease patterns.

Cluster analyses categorized the 21 GBD regions into four clusters based on the age‐standardized EAPC values for incidence, prevalence, mortality, and DALY associated with LOC over the past 32 years (Table S6). Western Europe and high‐income North America exhibited significant decreases or remained stable with minor decreases in ASPRs (Figure 6B) and ASIRs (Figure 6A) of LOC during this period. Central Europe, Eastern Europe, Southern Sub‐Saharan Africa, Eastern Sub‐Saharan Africa, Tropical Latin America, Southeast Asia, and the Caribbean formed a cluster of significant decrease in ASIRs (Figure 6A), ASMRs (Figure 6C), and ASDRs (Figure 6D) from 1990 to 2021. Moreover, this cluster was also a part of the cluster of remained stable or minor decrease ASPRs of LOC. Consistent with the above results, South Asia experienced significant increases in ASMRs and ASDRs, alongside a minor increase in ASIRs. Australasia exhibited a significant increase in ASPRs from 1990 to 2021.

**FIGURE 7:**
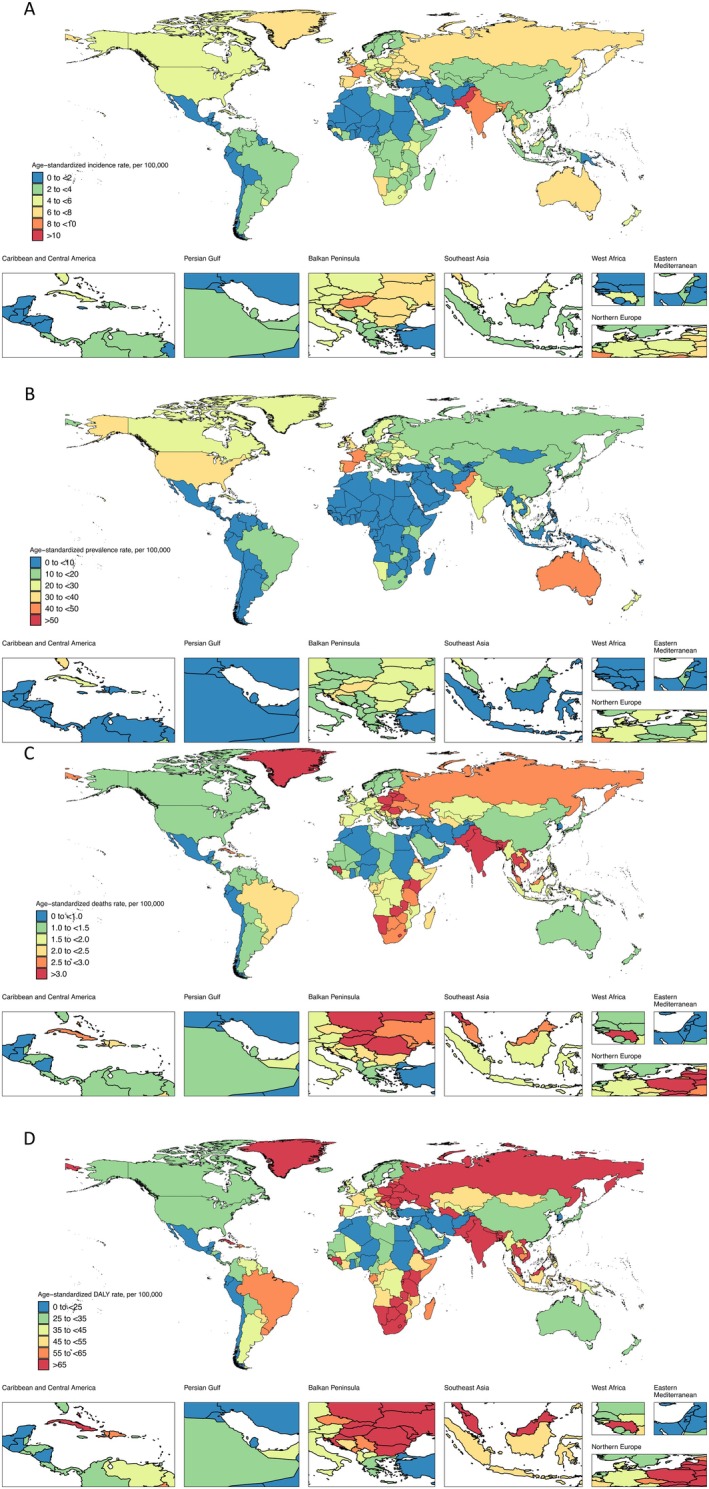
Global maps of the burden of lip and oral cavity cancer in 2021. In 2021, Eastern Europe, as well as South, North, and Southeast Asia, exhibited a concentration of countries with higher ASIRs, ASPRs, ASMRs, and ASDRs. South Asia displayed consistently high levels of ASIRs, ASPRs, ASMRs, and ASDRs in 2021. ASDR, age‐standardized disability rate; ASIR, age‐standardized incidence rate; ASMR, age‐standardized mortality rate; ASPR, age‐standardized prevalence rate.

Figure 7(A–D) presents the distribution maps of national‐level ASIRs, ASPRs, ASMRs, and ASDRs related to LOC in 2021. Countries in Eastern Europe and South, North, and Southeast Asia demonstrated a concentration of higher ASIRs, ASPRs, ASMRs, and ASDRs in 2021. South Asia exhibited uniformly high levels of ASIRs, ASPRs, ASMRs, and ASDRs in 2021. Detailed national‐level burden data for LOC is provided in Table S7. Among the countries, Palau, situated in Southeast Asia, had the highest ASIR in 2021, with a rate of 26.52 (95% UI, 20.51–33.37) incident cases per 100,000, as well as the highest ASPR [91.65 (95% UI, 70.34–117.48) prevalent cases per 100,000], ASMR [15.48 (95% UI, 11.99–19.65) mortalities per 100,000], and ASDR [432.66 (95% UI, 329.61–558.56) DALYs per 100,000]. Pakistan, located in South Asia, reported the second highest ASIR, ASPR, ASMR, and ASDR in 2021 (Table S7). Cabo Verde exhibited the most significant increases in incidence, prevalence, mortalities, and DALYs associated with LOC, showing the highest age‐standardized EAPCs from 1990 to 2021 (Table S7). In contrast, France recorded the largest decreases in mortalities and DALYs attributed to LOC, with the lowest age‐standardized EAPCs during these 32 years.

### 3.3 Prediction of Incidence, Prevalence, Mortality, and DALY of LOC

**FIGURE 8:**
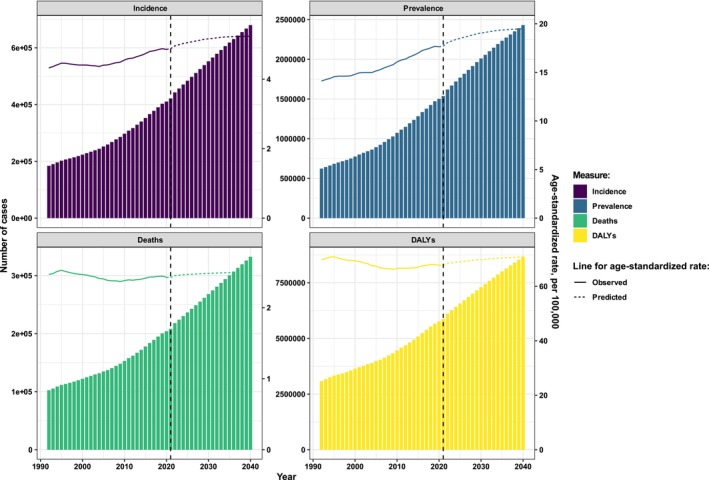
The observed and predicted results of the incidence, prevalence, mortality, and DALY of lip and oral cavity cancer from 1992 to 2040. The bar graph illustrates the number of cases related to incidence, prevalence, mortality, and DALY of lip and oral cavity cancer from 1992 to 2040. The curve line displayed the age‐standardized rate of incidence, prevalence, mortality, or DALY of lip and oral cavity cancer from 1992 to 2040. DALY, disability‐adjusted life years.

The overall trends for incidence, prevalence, mortalities, and DALYs exhibited significant increases over the observed timeframe, with predictions indicating continued growth over the next 20 years (Figure 8). The ASIRs and ASPRs of LOC are projected to show upward trends from 2021 to 2040, while the trends for ASMRs and ASDRs are expected to remain relatively stable during this period. Specifically, the number of cases of incidence, prevalence, mortalities, and DALYs was predicted to separately increase 61.31%, 58.06%, 58.53%, and 47.59% between 2021 and 2040 (Table S8). Furthermore, the ASIR, ASPR, ASMR, and ASDR are anticipated to rise by 7.40%, 10.10%, 2.85%, and 4.60%, respectively, between 2021 and 2040 (Table S8).

## 4 Discussion and Conclusion

This was the first study to comprehensively assess and quantify the global burden of LOC and then predict its future tendency using the GBD 2021 study. From 1990 to 2021, an upward trend was identified in ASPRs globally throughout the 32‐year period. The global trends in ASIRs, ASMRs, and ASDRs for LOC can be summarized as follows: an increase from 1990 (or 1991) to 1995, followed by a decline from 1995 to 2004 (or 2009), and then a significant increase from 2004 (or 2009) to 2019. Notably, a decline in ASIR, ASPR, ASMR, and ASDR was observed after 2019, with the decreasing trends in ASMR and ASDR continuing persistently into 2021. This may be attributed to the risk factors like tobacco [22, 23] and alcohol prevalence [24] for the same time period. For example, high smoking rates in the early 1990s, particularly among men, gradually declined due to the implementation of public health policies aimed at reducing tobacco use [22]. Consequently, there was a decrease in the level 3 risk‐attributable DALYs related to smoking from 2000 to 2021, which constitutes a significant component of tobacco‐related risk [23]. This decline also contributed to a reduction in mortality rates associated with diseases caused by smoking. Similarly, alcohol consumption initially increased in 2000–2006 but later declined as a result of awareness campaigns and public health interventions in 2006–2018 [24]; nonetheless, some regions saw a subsequent rise in alcohol consumption.

Consistent with findings from other studies [2, 25], males consistently exhibited higher numbers of reported cases and ASIRs, ASPRs, ASMRs, and ASDRs of LOC compared to females. This may be attributed to the consistent increase in the male population relative to females over the past 32 years, potentially contributing to the rising incidence and mortality rates of LOC among males (https://data.worldbank.org/indicator/SP.POP.TOTL.FE.ZS?end=2023&start=1960&view=chart). However, females demonstrated greater variation in EAPC from 1990 to 2021. In 2021, the population aged 60–64 years comprised the highest number of new and prevalent cases. The ASIR, ASPR, ASMR, and ASDR were highest among elderly people in both 2019 and 2021. The increasing elderly population significantly contributes to the rise in cases, particularly among those aged 60–95+ years. From 1990 to 2021, low‐middle and low SDI regions consistently recorded higher ASMRs and ASDRs attributed to LOC. Conversely, high and high‐middle SDI regions displayed a decreasing trend in both ASMR and ASDR. Both high and low‐middle SDI regions exhibited elevated ASIRs and ASPRs over the past 32 years. The observed differences in ASMRs for LOC within regions possessing similar ASIRs suggest that individuals in high SDI regions may experience better survival outcomes from LOC, potentially attributable to earlier‐stage diagnoses and/or effective disease management and intervention strategies [26, 27, 28, 29].

According to the results from the 21 regions included in the GBD 2021 study, South Asia consistently reported the highest numbers and ASRs of incidence, mortality, and DALYs in 1990, 2019, and 2021, alongside a general upward trend in these ASRs. Previous studies have reported that chewing tobacco remains a significant public health issue in various regions around the world, particularly in South Asia [30]. Meanwhile, Australasia exhibited the highest ASPRs and a significant increasing trend in ASPRs from 1990 to 2021. Eastern Europe, as well as South, North, and Southeast Asia, displayed a concentration of countries with higher ASIRs, ASPRs, ASMRs, and ASDRs in 2021. Similarly, two Eastern European countries (Belarus and Moldova) separately had the highest in ASIR and ASMR in the Global Cancer Observatory (GLOBOCAN) [31] 2017 database. These findings underscore the necessity for targeted health policies and interventions in regions with a high burden of disease, particularly in Eastern Europe, South, North, and Southeast Asia. Notably, a previous study indicated that approximately 83.29% (95% UI, 82.15%–84.42%) of chewing tobacco users resided in South Asia in 2019 [30], highlighting a significant public health concern that may contribute to the higher incidence, mortality, and DALY rates associated with LOC.

In the GBD 2017 study, Pakistan was identified as having the highest ASIR of oral cancer at 27.03 per 100,000 individuals (95% CI = 22.13–32.75) [3]. Consistent with the findings, Pakistan also exhibited the second highest ASIR of LOC in 2021. Additionally, we found that Palau reported the highest ASIR, ASPR, ASMR, and ASDR of LOC in 1990, 2019, and 2021. Both Palau and Pakistan are recognized as countries with a high prevalence of chewing tobacco use [30]. Similarly, betel quid and areca nut chewing is prevalent among Asian Pacific Islander (i.e., Palau, etc.) [32] and South Asian (i.e., Pakistan, etc.) [33] adults, with adding tobacco to the chew. The use of chewing or other smokeless tobacco may lead to various oral health issues, including erythroplakia, leukoplakia, periodontal support loss, and oral submucous fibrosis [34]. Prolonged use significantly increases the risk of oral cancer [35].

The overall trends in the incidence, prevalence, mortality, and DALYs associated with loss of LOC have demonstrated a significant increase during the observed period and are projected to continue rising over the next 20 years. The ASIRs and ASPRs for LOC are expected to exhibit upward trends from 2021 to 2040, while the ASMRs and ASDRs are anticipated to remain relatively stable during this period. Specifically, from 2021 to 2040, the ASIR is predicted to increase by 7.40%, the ASPR by 10.10%, the ASMR by 2.85%, and the ASDR by 4.60%. Therefore, there is a critical need to focus on oral health globally, particularly in high‐burden genders, age groups, regions, and countries. It is also recommended that a series of public health interventions targeting LOC globally, including strengthening tobacco control initiatives through telephone quit lines and anti‐tobacco education in schools to raise awareness among youth, be implemented. Additionally, increasing taxes on tobacco products under the WHO MPOWER initiative is suggested to reduce consumption. Early detection and prevention of LOC are deemed crucial, with recommendations for community‐level screening programs to lower related infection rates.

This study presents several strengths, including a comprehensive summary of disease burden data related to LOC from the GBD database. By incorporating the latest data from the GBD 2021 study, it significantly advances the understanding of LOC‐related trends compared to prior research. Notably, while global ASMRs and ASDRs associated with LOC have decreased, the projections for the coming decades raise important concerns, indicating a potential increase in these rates from 2021 to 2040. Moreover, the study highlights the pressing need for urgent interventions to enhance public health strategies, particularly for vulnerable populations. These include males, individuals aged 60–64 years and those aged 95 years and older, as well as residents of low‐ and middle‐SDI regions, such as Eastern Europe, South Asia, North Asia, and Southeast Asia. Future research could enhance these findings by employing multi‐dataset comparisons and presentations, providing a more detailed illustration, and calculating the cumulative rates of the global burden of LOC.

## Author Contributions

**Mingxing Chen:** conceptualization, methodology, software, validation, investigation, writing – original draft, data curation, visualization. **Jiangxi Li:** investigation, writing – review and editing. **Wei Su:** investigation, supervision, writing – review and editing. **Junming Huang:** visualization, writing – review and editing. **Chuanzhen Yang:** methodology, software, visualization. **Rui Li:** methodology, software, visualization. **Gang Chen:** conceptualization, formal analysis, writing – review and editing, writing – original draft.

## Ethics Statement

The authors have nothing to report. This study is a bibliometric analysis, which does not contain any studies with humans or animals performed.

## Consent

The authors have nothing to report.

## Conflicts of Interest

The authors declare no conflicts of interest.

## Supporting information


Data S1.

**Figure S1:** The flow chart of the global burden of LOC study using the GBD 2021 data. ASDR, age‐standardized disability rate; ASIR, age‐standardized incidence rate; ASMR, age‐standardized mortality rate; ASPR, age‐standardized prevalence rate; EAPC, estimated annual percentage change.

**Table S1.** The incidence, prevalence, deaths, and DALYs of lip and oral cavity cancer in 1990, 2019, 2021 and its trend from 1990 to 2021 globally.
**Table S2.** The incidence, prevalence, deaths, and DALYs of lip and oral cavity cancer of different genders in 1990, 2019, 2021 and its trend from 1990 to 2021.
**Table S3.** The incidence, prevalence, deaths, and DALYs of lip and oral cavity cancer of different age groups in 1990, 2019, and 2021.
**Table S4.** The incidence, prevalence, deaths, and DALYs of lip and oral cavity cancer of different SDI locations in 1990, 2019, 2021 and its trend from 1990 to 2021.
**Table S5.** The incidence, prevalence, deaths, and DALYs of lip and oral cavity cancer of 21 regions in 1990, 2019, and 2021.
**Table S6.** The results of the incidence, prevalence, deaths, and DALYsof lip and oral cavity cancer in 21 regions using K_mean analysis.
**Table S7.** The incidence, prevalence, deaths, and DALYs of lip and oral cavity cancer of 204 countries in 1990, 2019, 2021, and its trend from 1990 to 2021.
**Table S8.** The observed and predicted results of the incidence, prevalence, deaths, and DALYs of lip and oral cavity cancer from 1992 to 2040.

## Data Availability

The dataset supporting the conclusions of this article can be requested from the corresponding author.
