# Correction to Global Burden of Lip and Oral Cavity Cancer From 1990 to 2021 and Projection to 2040: Findings From the 2021 Global Burden of Disease Study

**DOI:** 10.1002/cam4.71006

**Published:** 2025-06-29

Chen, M., Li, J., Su, W., Huang, J., Yang, C., Li, R. and Chen, G. (2025), Global Burden of Lip and Oral Cavity Cancer From 1990 to 2021 and Projection to 2040: Findings From the 2021 Global Burden of Disease Study. *Cancer Med*, 14: e70957. https://doi.org/10.1002/cam4.70957.

The address information of the first author, Mingxing Chen, “^1^Medical Research Public Service Center, The First Affiliated Hospital, Jiangxi Medical College, Nanchang University, Beijing, China” was filled in incorrectly. The correct address should be “^1^Medical Research Public Service Center, The First Affiliated Hospital, Jiangxi Medical College, Nanchang University, Nanchang, China”.

We apologize for this error.

