# Supplementary material for: Global Burden of Lip and Oral Cavity Cancer From 1990 to 2021 and Projection to 2040: Findings From the 2021 Global Burden of Disease Study
Source: Cancer Med. 2025 May 10;14(9):e70957. doi: 10.1002/cam4.70957 (PMC12065076; doi:10.1002/cam4.70957)
Supplement: Supplementary file 2 — Figure S1: The flow chart of the global burden of LOC study using the GBD 2021 data. ASDR, age‐standardized disability rate; ASIR, age‐standardized incidence rate; ASMR, age‐standardized mortality rate; ASPR, age‐standardized prevalence rate; EAPC, estimated annual percentage change. [file CAM4-14-e70957-s002.pdf]

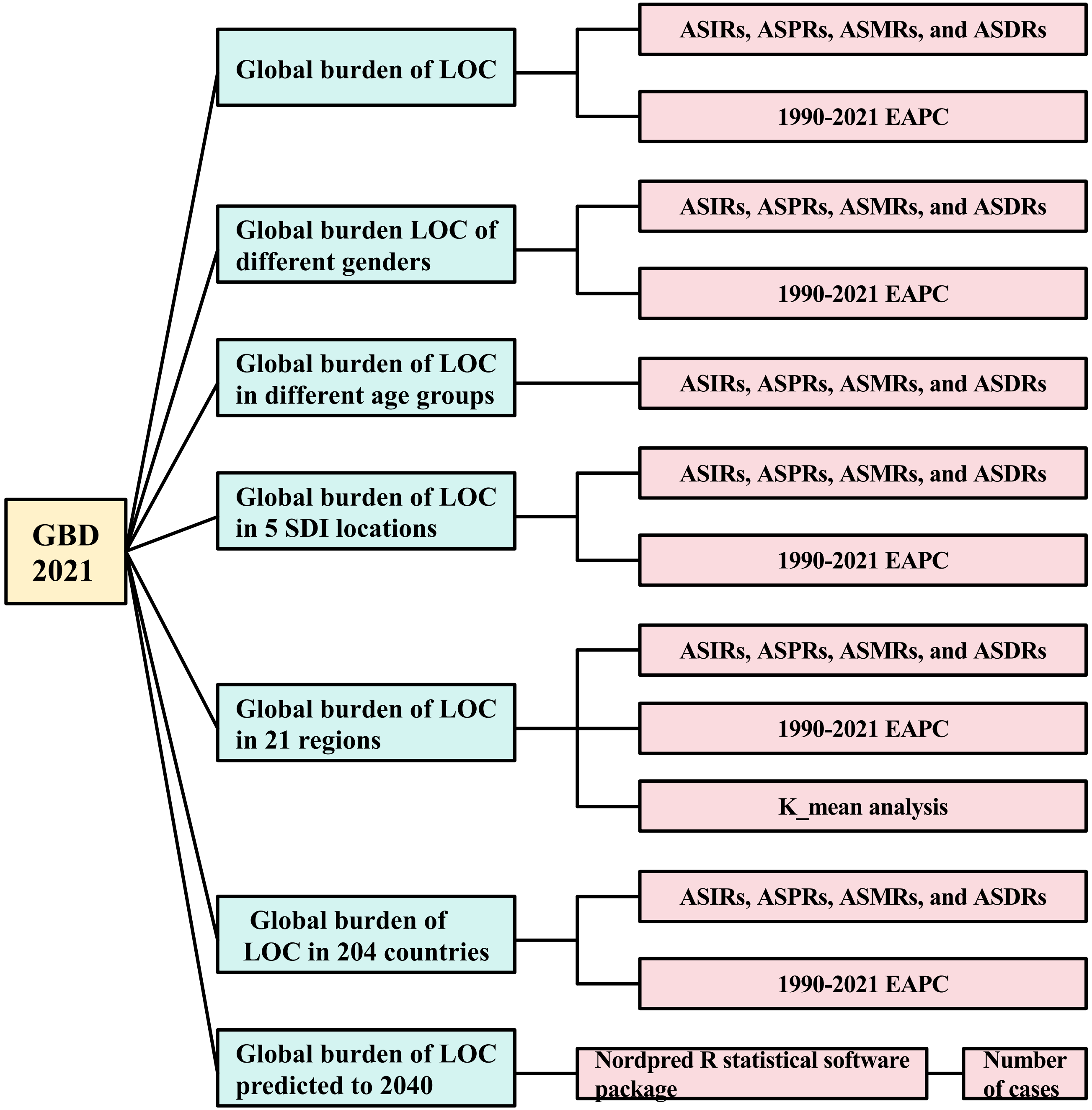

Figure S1. The flow chart of the global burden of LOC study using the GBD 2021 data.
ASIR, age-standardized incidence rate; ASPR, age-standardized prevalence rate; ASMR, age-standardized mortality rate; ASDR, age-standardized disability rate; EAPC, estimated annual percentage change.
